# The Influence of Helium Dielectric Barrier Discharge Jet (DBDjet) Plasma Treatment on Bathocuproine (BCP) in p-i-n-Structure Perovskite Solar Cells

**DOI:** 10.3390/polym13224020

**Published:** 2021-11-20

**Authors:** Chung-Yueh Shih, Jian-Zhi Huang, Mei-Hsin Chen, Cheng-Che Hsu, Chih-I Wu, I-Chun Cheng, Jian-Zhang Chen

**Affiliations:** 1 Graduate Institute of Applied Mechanics, National Taiwan University, Taipei City 10617, Taiwan; r09543015@ntu.edu.tw; 2 Advanced Research Center for Green Materials Science and Technology, National Taiwan University, Taipei City 10617, Taiwan; 3 Graduate Institute of Photonics and Optoelectronics, National Taiwan University, Taipei City 10617, Taiwan; f07941064@ntu.edu.tw (J.-Z.H.); chihiwu@ntu.edu.tw (C.-I.W.); iccheng@ntu.edu.tw (I.-C.C.); 4 Department of Electro-Optical Engineering, National Taipei University of Technology, Taipei City 10608, Taiwan; mhchen@mail.ntut.edu.tw; 5 Department of Chemical Engineering, National Taiwan University, Taipei City 10617, Taiwan; chsu@ntu.edu.tw; 6 Department of Electrical Engineering, National Taiwan University, Taipei City 10617, Taiwan; 7 Innovative Photonics Advanced Research Center (i-PARC), National Taiwan University, Taipei City 10617, Taiwan

**Keywords:** atmospheric-pressure plasma, dielectric barrier discharge, perovskite solar cell, conductive polymer, non-thermal plasma

## Abstract

A bathocuproine (BCP) layer is typically used as the hole-blocking layer in p-i-n-structure perovskite solar cells (PSCs) between PC_61_BM and Ag electrodes. Before evaporating the Ag, we used a low-temperature (<40 °C) atmospheric-pressure dielectric barrier discharge jet (DBDjet) to treat the BCP with different scan rates. The main purpose of this was to change the contact resistance between the BCP layer and the Ag electrodes through surface modification using a DBDjet. The best power conversion efficiency (PCE) of 13.11% was achieved at a DBDjet scan rate of 2 cm/s. The He DBDjet treatment introduced nitrogen to form C−N bonds and create pits on the BCP layer. This deteriorated the interface between the BCP and the follow-up deposited-Ag top electrode. Compared to the device without the plasma treatment on the BCP layer, the He DBDjet treatment on the BCP layer reduced photocurrent hysteresis but deteriorated the fill factor and the efficiency of the PSCs.

## 1. Introduction

In the past few years, organic-inorganic hybrid perovskite solar cells (PSCs) have gained attention due to their high power conversion efficiency (PCE) and low cost [1,2,3,4,5,6,7,8,9]. In 2013, the first p-i-n-structure PSC was fabricated and it had a PCE of 3.9% [10]. Nowadays, p-i-n-structure PSCs with PCEs exceeding 22% are being fabricated [11].

A conventional p-i-n structure-PSC comprises a hole transport layer (HTL), a perovskite, and an electron transport layer (ETL). The contact resistance between the PCBM and the metal is due to the energy band mismatch. To reduce the charge combination, bathocuproine (BCP), LiF, and ZnO are commonly used as nanometer-thick buffer layers that are placed between the ETL and the metal electrode. For instance, Seo et al., optimized the [6,6]-phenyl-C61-butyric acid methyl ester (PCBM) thickness through the insertion of a LiF layer in PSCs and fabricated a device with a PCE exceeding 14%. Qiu et al. also found that the PSC with a PCBM/ZnO bilayer stored in air for a short duration exhibited a better device performance [12,13,14,15,16,17,18,19,20,21,22]. Chen et al. noted that a BCP buffer layer reduces charge accumulation and recombination at the ETL/metal electrode interface; furthermore, they found that a BCP buffer layer of suitable thickness not only results in excellent photovoltaic (PV) performance, but also provides an encapsulation effect that enhances the device stability [23]. The use of BCP in PSCs has been widely investigated. However, the surface modification of BCP with plasma treatment in a PSC system has not yet been reported.

Atmospheric-pressure plasma (APP) can be operated at a regular pressure without using a vacuum system. Typical APP technologies include corona discharge and dielectric barrier discharge (DBD) with or without a high-speed jet rate for cooling and for bringing out the reactive plasma species [24,25,26]. In biomedicine [27,28,29] and agriculture [30,31] applications, low-temperature APP has been extensively used because it can avoid thermal damage. In other applications, the effects of heat and reactive plasma species can be synergized to realize the ultrafast processing of materials [32,33,34,35]. Atmospheric-pressure plasma has been used for processing PSCs in various ways. Hilt et al. converted sprayed perovskite precursor films using a plasma jet [36]. Ameen et al. processed the ZnO quantum dots ETL of PSCs on a flexible substrate [37]. Homola et al. used air plasma to process mesoporous TiO_2_ for PSCs made on a glass substrate [38].

**Figure 1:**
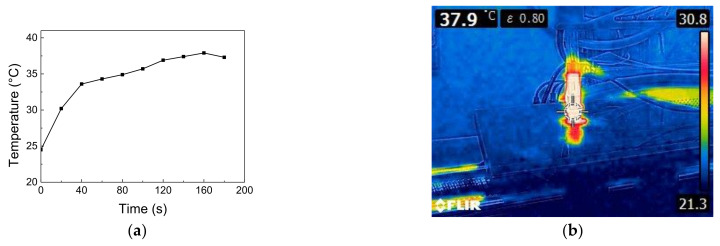
(**a**) Working temperature and (**b**) infrared thermal image during DBDjet processing.

Our research group has worked on the APP processing of PSCs for several years. Previously, we used a portable surface-diffusion DBD device to treat the perovskite layer for both p-i-n and n-i-p PSCs. With the use of a proper processing time, the efficiency of PSCs can be improved [39,40]. The same surface-diffusion DBD device was also applied to treat the PEDOT:PSS film of PSCs [41]. A 500 °C arc APP jet was used to post-treat the NiO HTL of the PSCs to improve their performance [42]. A <40 °C helium (He) DBDjet was also used for treating the NiO HTL of the PSCs for better performance [43]. The same DBDjet was also used for stripping the polyvinylpyrrolidone outside the jet-sprayed Ag nanowire electrodes of fully solution-processed n-i-p structure PSCs to improve the device performance [44]. Most recently, this DBDjet was also used to treat low-temperature TiO_2_ nanoparticle ETLs of PSCs on a polyethylene naphthalate substrate [45]. In this study, we used a scan-mode He DBDjet to treat the BCP layer of p-i-n PSCs (FTO/NiO/perovskite/PC_61_BM/BCP/Ag) with different scan rates (3, 2, 1, and 0.5 cm/s) and investigated the influence of the He DBDjet treatment on the BCP layer. An inverted PSC with a BCP layer was found to degrade at temperatures higher than 85 °C. Hence, in this work, the He DBDjet plasma treatment on the BCP layer was performed while keeping the working temperature below 40 °C (Figure 1), thereby avoiding thermal damage [46,47]. Therefore, the major effect resulted from the reactive plasma species. Furthermore, we investigated the performance of PSCs in which the BCP layer was treated using the He DBDjet before the Ag electrode deposition.

## 2. Materials and Methods

### 2.1. PSC Fabrication

A 2 cm × 2 cm fluorine-doped tin oxide (FTO, TEC7, sheet resistance: ~8 Ω sq ^−1^, SCiKET, Taoyuan, Taiwan) glass substrate was sequentially cleaned using deionized water, acetone, isopropanol, and a UV-ozone cleaner for 15 min. A NiO precursor solution was prepared by dissolving 0.5 M nickel acetate (99.998%, trace metals basis, Sigma-Aldrich, St. Louis, MO, USA) and ethanolamine (99.5%, Sigma-Aldrich, St. Louis, MO, USA) in ethanol (99%, anhydrous alcohol, Echo Chemical Co. Ltd., Miaoli, Taiwan) and stirring overnight at 60 °C. Then, the NiO precursor solution was spin-coated on the FTO substrate at 6000 rpm for 40 s and calcined on the hot plate at 325 °C for 10 min. The resulting NiO layer served as the HTL. The sample was then immediately transferred into a nitrogen-filled glove box. The perovskite precursor solution was prepared by dissolving 578 mg of PbI_2_ (99.999%, metals basis, Alfa Aesar, MA, USA) and 200 mg of CH_3_NH_3_I (MAI, 98%, UniRegion Bio-Tech, Taoyuan, Taiwan) in 1 mL of dimethylformamide (99.8%, Sigma-Aldrich, St. Louis, MO, USA) with stirring at 500 rpm at 60 °C for 12 h. The perovskite film was deposited on the NiO film through a one-step method. The resulting perovskite film was annealed at 100 °C for 10 min. Then, 1 mL of PC_61_BM (99.5%, UniRegion Bio-Tech, Taoyuan, Taiwan) doped with 2 μL of DMOAP (42% methanol solution, Sigma-Aldrich, St. Louis, MO, USA) was spin-coated on the perovskite film at 2000 rpm for 30 s. Next, BCP (0.5 mg mL^−1^ in 2-propano, Alfa Aesar, MA, USA) was spin-coated on the PC_61_BM film at 6000 rpm for 20 s and then annealed at 75 °C for 15 min. Then, the BCP was scanned once by He DBDjet plasma at scan rates of 0.5, 1, and 2 cm/s. The gap between the sample and the bottom of the quartz tube was fixed at 1 mm in the He DBDjet treatment process. Finally, Ag (99.99%, Gredmann, Taipei, Taiwan) electrodes were deposited by e-beam evaporation. The thickness of the Ag layer was 85 nm. These electrodes (specifically, cathodes) were controlled to have an area of 0.09 cm^2^ (0.3 cm × 0.3 cm) on each cell via a shadow mask. The He DBDjet-scanned samples were then compared with the untreated sample.

### 2.2. Characterization

The surface morphology of BCP was inspected using scanning electron microscopy (SEM, JSM-7800F Prime, JEOL, Tokyo, Japan). The surface chemical bonding status was surveyed by a custom-made X-ray photoelectron spectroscopy (XPS) system (PHI 5400 XPS system, PHI, Chanhassen, MN, USA). The external quantum efficiency (EQE) of the PSCs was measured using a quantum efficiency analyzer (Enlitech, QE-R3011, Kaohsiung, Taiwan). The electrochemical performance was evaluated by electrochemical impedance spectroscopy (EIS) using an electrochemical workstation (Autolab PGSTAT204, Metrohm, Utrecht, The Netherlands). The current density–voltage (J–V) curve of the PSC was measured using a sourcemeter (B2902A, Agilent, Santa Clara, CA, USA) under the illumination of simulated AM1.5 light (Sun 2000 Solar Simulator, ABET, Milford, CT, USA).

## 3. Results and Discussion

### 3.1. SEM Analysis

**Figure 2:**
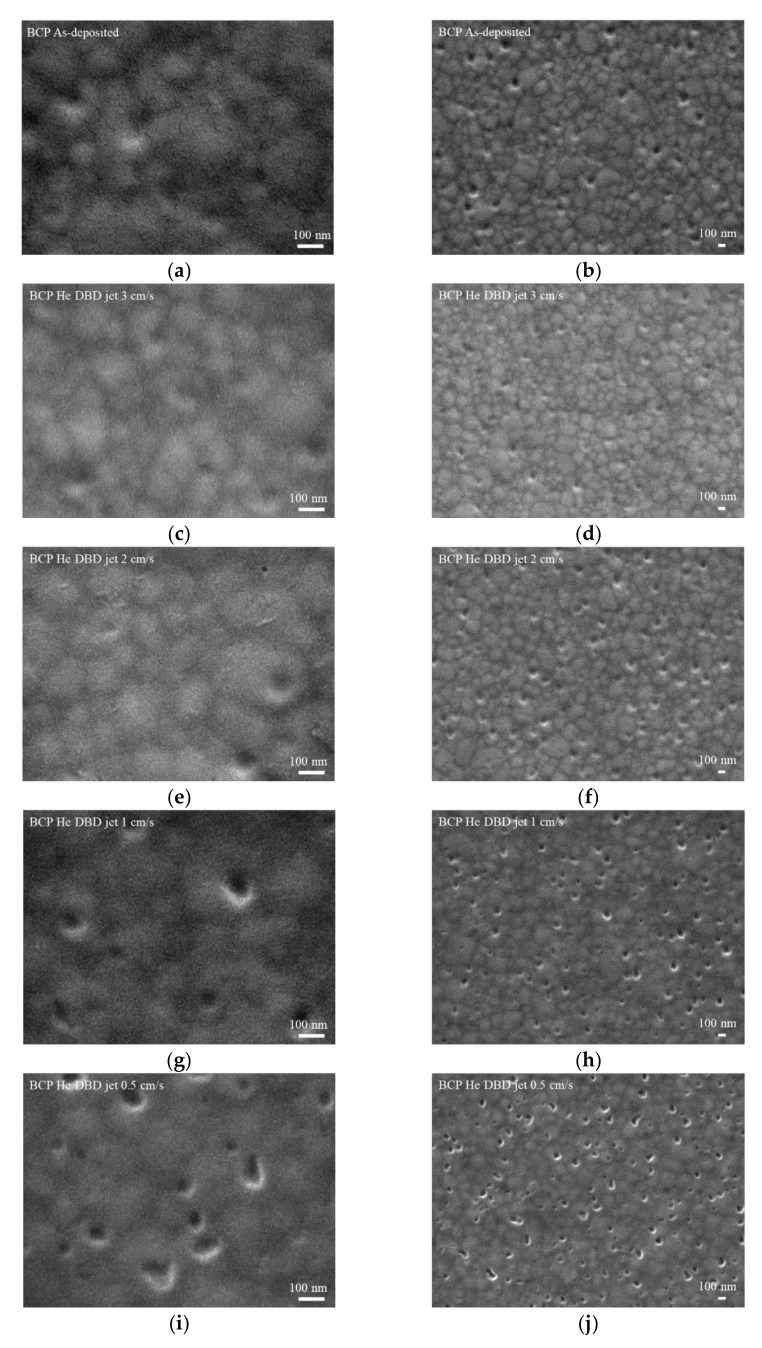
Top-view SEM images of BCP films (**a**,**b**) without and (**c**–**j**) with DBDjet treatment under scan rates of (**c**,**d**) 3, (**e**,**f**) 2, (**g**,**h**) 1, and (**i**,**j**) 0.5 cm/s.

Figure 2 demonstrates surface images of the BCP films without and with the He DBDjet plasma treatment. After the He DBDjet treatment on the BCP layer at a lower scan rate, some pits could be clearly seen on the BCP layer. Further, the number of pits increased as the scan rate decreased. This suggests that the BCP layer likely suffered surface oxidation and damage during the plasma treatment.

### 3.2. XPS Analysis

**Figure 3:**
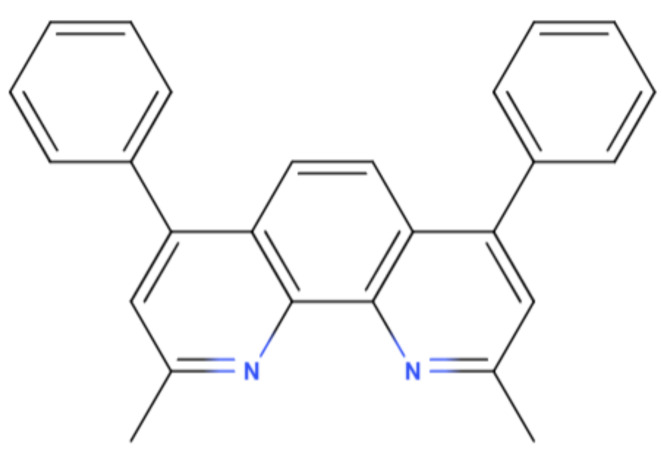
Structure of BCP.

**Figure 4:**
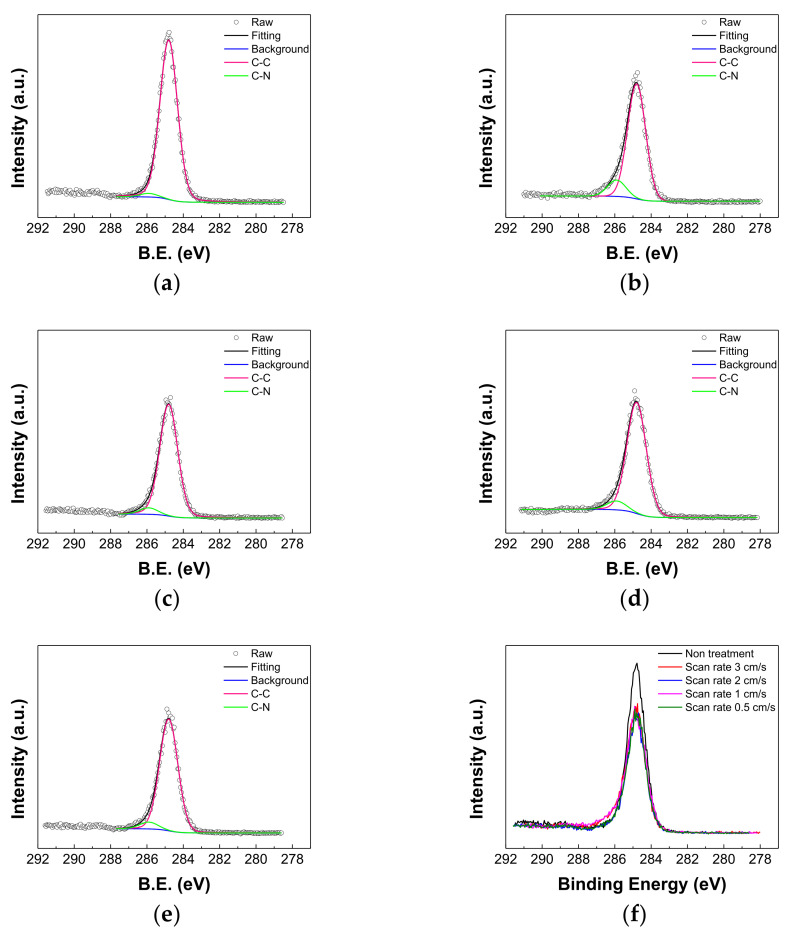
XPS of C1s spectra of BCP film (**a**) as-deposited and (**b**–**e**) with He DBDjet treatment with scan rates of (**b**) 3, (**c**) 2, (**d**) 1, and (**e**) 0.5 cm/s. (**f**) Superimposed XPS C1s spectra.

All binding energies were referenced to C1s (284.8 eV). Figure 3 shows the structure of BCP. Figure 4 shows the XPS C1s spectra of the BCP film with and without the He DBDjet treatment and a superimposed XPS C1s spectra. The peaks at 284.8 and 285.9 eV corresponded to C–C and C–N, respectively [48]. Table 1 lists the contents of the corresponding deconvolution peaks. Owing to the reaction between the BCP and the reactive plasma species of the He DBDjet, the intensity of the C–C binding energy peak decreased, as seen in Figure 4f. Then, under the He DBDjet treatment, a lower scan rate resulted in a decrease in the proportion of C–N bonds, as shown in Table 1. However, the amounts of C−N bonds in the samples with the DBDjet scanning were all larger than in those without the DBDjet treatment. This suggests that the environmental nitrogen may participate in the plasma reaction and introduce nitrogen species to the sample. This is evidenced in our previous studies regarding the processing of DBDjet materials [43,49]; reactive nitrogen species are clearly identified in the optical emission spectra of the He DBD jet plasma. These reactive nitrogen species may react with the BCP to form C–N bonds in the first instance. With a higher plasma processing time—i.e., a smaller DBDjet scan rate—the amount of C–N bonds will be lower in turn, possibly owing to the more vigorous plasma reaction or damage to the BCP layer. From the results shown in Figure 4 and Table 1, it may be reasonable to assume that the reactive plasma species in the He DBDjet reacted with the BCP to produce pits (i.e., damage), as seen in the SEM results.

### 3.3. External Quantum Efficiency Analysis

**Figure 5:**
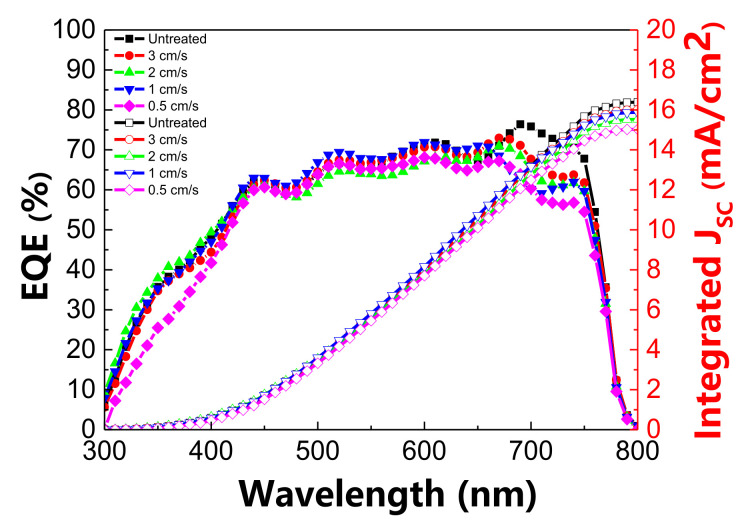
EQE and corresponding integrated photocurrent curves of p-i-n PSCs with He DBDjet treatment on the BCP layer.

The EQE and corresponding integrated photocurrent curve of the p-i-n-structure PSCs with the He DBDjet treatment on the BCP film are illustrated in Figure 5. The results indicate that the He DBDjet treatment slightly deteriorated the EQE and the corresponding integrated photocurrent. The wavelength range of 650 to 750 nm had the greatest influence. The integrated photocurrent decreased from 16.387 (without DBDjet treatment) to 15.031 mA cm^−2^ (with DBDjet treatment at the lowest scan rate of 0.5 cm/s). At the lowest scan rate of 0.5 cm/s, the plasma influencing time is longer; therefore, the EQE degradation was more apparent. Additional degradation apparently occurred in the wavelength range of 300–400 nm at a DBDjet scan rate of 0.5 cm/s. The EQE possibly decreased due to more interface charge accumulation or radiative recombination [13,23]. Because the DBDjet plasma working temperature was below 40 °C, this result was mainly attributed to the reactive plasma species.

### 3.4. Electrochemical Impedance Spectroscopy Analysis

**Figure 6:**
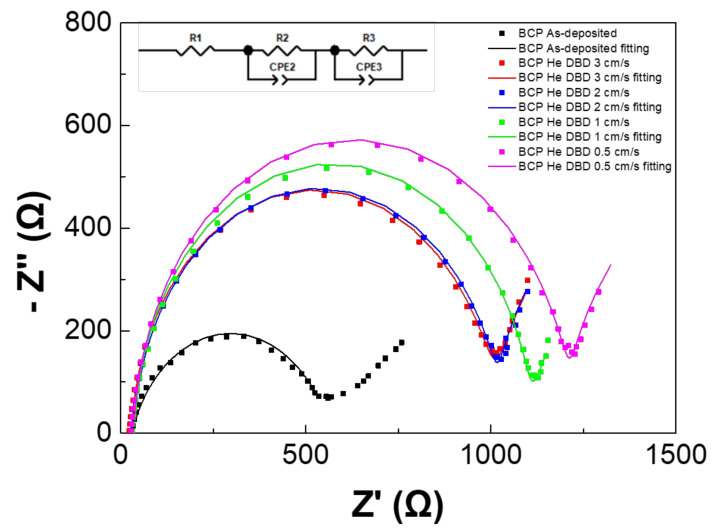
The Nyquist plot of EIS.

Figure 6 shows a Nyquist plot of the EIS. Its inset shows the equivalent circuit for analyses. Further, Table S1 lists the EIS fitting parameters. R1, R2, and R3 correspond to the series resistance, charge transporting resistance, and recombination resistance, respectively [42,50]. Because the same FTO substrates and top electrodes were used for the devices, the R1 is almost the same for the devices. A smaller R1 and R2 could provide benefits through the encouragement of photogenerated carriers and transportation to the corresponding HTL and ETL [51]. The R2 increased (higher charge transporting resistance) after the He DBDjet treatment on the BCP layer. More specifically, the R2 monotonically increased with a decrease in the He DBDjet scan rate. This suggests that the carrier transport between the BCP layer and the Ag was degraded [42] after the DBDjet treatment.

### 3.5. Statistical Analysis of PV Parameters

**Figure 7:**
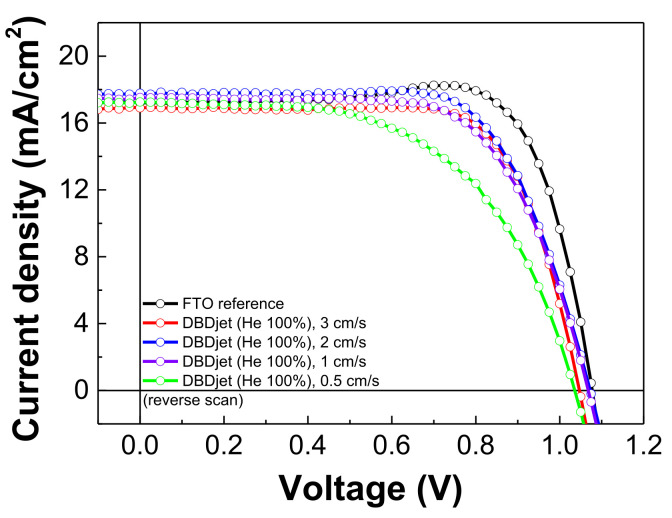
J–V curves of PSCs with He DBDjet treatment on the BCP layer (reverse scan).

Figure 7 presents the J–V curves of the PSCs with the He DBDjet treatment on the BCP layer at different scan rates (reverse scan). Table 2 lists the corresponding photovoltaic parameters. The PV parameters include the open circuit voltage (V_oc_), short circuit current density (J_sc_), fill factor (F.F.), and PCE. The results show that the use of plasma treatment (i.e., with a smaller DBDjet scan rate) on the BCP layer for a longer time deteriorated the PSCs’ performance. Compared to the PSCs without the He DBDjet treatment, the PSCs with the He DBDjet treatment exhibited smaller hysteresis, defined as (PCE (forward scan)—PCE (reverse scan))/PCE (forward scan), as shown in Figure S1 [4,52,53]. Many parameters influence the J–V hysteresis behavior of the PSCs, such as the voltage range, scan direction, scan rate, architecture, and precondition of PSCs [54,55,56,57,58,59,60,61,62,63]. In this case, without the He DBDjet treatment on the BCP layer, the unbalanced electron and hole transport rates as well as interface defects may cause photocurrent hysteresis [4,52,53]. Figures S2 and S3, and Table S2 show the statistical data for five batches of PSCs with He DBDjet treatment on the BCP. No apparent degradation was found in V_oc_ or J_sc_, and the results show that a longer plasma treatment time on the BCP led to a lower F.F., in keeping with the SEM results. The pits on the BCP layer led to an unsmooth film morphology, in turn resulting in a low F.F. and a low EQE owing to the leakage current [64,65]. Further, the performance under a reverse scan was much poorer than that under a forward scan after He DBDjet treatment. Thus, the hysteresis of the PSC with He DBDjet treatment on the BCP reduced, as seen in Table S2.

## 4. Conclusions

This study investigated the influence of He DBDjet plasma treatment on a BCP layer. The best PCE of 13.11% was achieved in a PSC subjected to He DBDjet treatment at a scan rate of 2 cm/s. EQE and EIS results indicated that the He DBDjet treatment negatively affected the electronic states of BCP used as a HBL for PSCs. He DBDjet treatment introduced nitrogen to form C−N bonds and produce pits on the BCP layer. This deteriorated the interface of the BCP and the follow-up deposited-Ag top electrode. Compared with the reference cell (i.e., no plasma treatment), He DBDjet treatment on the BCP layer deteriorated the PSCs’ performance. In particular, it impacted the F.F. of the PSCs. Furthermore, the hysteresis of the PSC decreased after He DBDjet treatment on the BCP layer.

## Figures and Tables

**Table 1:** XPS deconvolution results for C1s spectra of the BCP film.

%	C–C	C–N
BCP as-deposited	97.81	2.19
BCP He DBDjet 3 cm/s	87.56	12.44
BCP He DBDjet 2 cm/s	94.55	5.45
BCP He DBDjet 1 cm/s	92.65	7.35
BCP He DBDjet 0.5 cm/s	94.24	5.76

**Table 2:** Photovoltaic parameters of PSCs without/with He DBDjet treatment on the BCP layer.

		V_oc_ (V)	J_sc_ (mA/cm^2^)	F.F. (%)	PCE (%)
No plasma treatment	Forward	1.07	16.63	64.71	11.46
	Reverse	1.08	17.21	78.60	14.62
Scan rate 3 cm/s	Forward	1.05	16.62	65.83	11.52
	Reverse	1.05	16.91	72.08	12.76
Scan rate 2 cm/s	Forward	1.07	17.33	62.50	11.57
	Reverse	1.07	17.78	68.72	13.11
Scan rate 1 cm/s	Forward	1.07	17.16	62.26	11.40
	Reverse	1.07	17.53	66.26	12.41
Scan rate 0.5 cm/s	Forward	1.05	16.88	53.01	9.36
	Reverse	1.04	17.25	56.20	10.06

## Data Availability

The relevant data are all included in the paper and in the Supplementary Materials.

## Supplementary Materials

The following are available online at www.mdpi.com/article/10.3390/polym13224020/s1: Figure S1: Hysteresis of PSCs without/with He DBDjet plasma treatment on BCP; Figure S2: PV parameters of PSCs with He DBDjet treatment on BCP (forward scan); Figure S3: PV parameters of PSCs with He DBDjet treatment on BCP (reverse scan); Figure S4. Cross-sectional view SEM images of the samples. Table S1: EIS fitting parameters; Table S2: PV parameters of PSCs with He DBDjet treatment on BCP.
