# Supplementary material for: The Influence of Helium Dielectric Barrier Discharge Jet (DBDjet) Plasma Treatment on Bathocuproine (BCP) in p-i-n-Structure Perovskite Solar Cells
Source: Polymers (Basel). 2021 Nov 20;13(22):4020. doi: 10.3390/polym13224020 (PMC8622915; doi:10.3390/polym13224020)
Supplement: Supplementary file 1 [file polymers-13-04020-s001.zip › polymers-1446326-supplementary.pdf]

(Supplementary Information)

Influence of Helium Dielectric-Barrier-Discharge-jet (DBDjet) Plasma Treatment on Bathocuproine (BCP) for p-i-n Structure Perovskite Solar Cells

Chung-Yueh Shih[1,7], Jian-Zhi Huang[4], Mei-Hsin Chen[2], Cheng-Che Hsu[3], Chih-I Wu[4], I-Chun Cheng[4,5,6], and Jian-Zhang Chen[1,6,7,*]

[1] *Graduate Institute of Applied Mechanics, National Taiwan University, Taiwan*

[2] *Department of Electro-Optical Engineering, National Taipei University of Technology, Taiwan*

[3] *Department of Chemical Engineering, National Taiwan University, Taiwan*

[4] *Graduate Institute of Photonics and Optoelectronics, National Taiwan University, Taiwan*

[5] *Department of Electrical Engineering, National Taiwan University, Taiwan*

[6] *Innovative Photonics Advanced Research Center (i-PARC), National Taiwan University, Taiwan*

[7] *Advanced Research Center for Green Materials Science and Technology, National Taiwan University*, Taiwan

**Email: jchen@ntu.edu.tw*

Table S1. EIS fitting parameters

PCB processing condition	R1 (Ω)	R2 (Ω)	CPE2-T (F)	CPE2-P	R3 (Ω)	CPE3-T (F)	CPE3-P
As-deposited	30	530	1E-7	0.8	150	5E-7	1.2
3 cm/s He DBD jet	29.3	940	6.31E-9	1	9000	1.32E-5	0.72
2 cm/s He DBD jet	35.69	946.4	6.40E-9	1	2588	5.62E-6	0.82
1 cm/s He DBD jet	37.34	1050	6.29E-9	1	1850	1.35E-5	0.81

0.5 cm/s He DBD jet	34.75	1145	6.21E-9	1	2500	7.2E-6	0.82

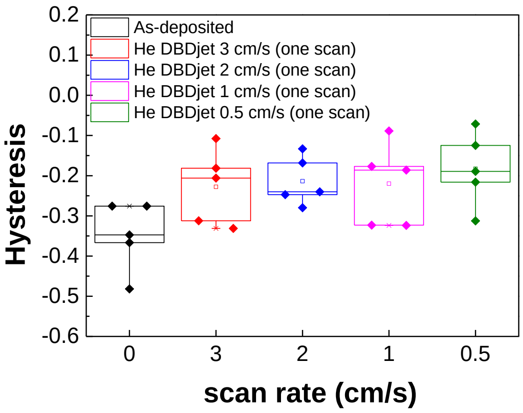

Figure S1. Hysteresis of PSCs without/with He DBDjet plasma treatment on BCP

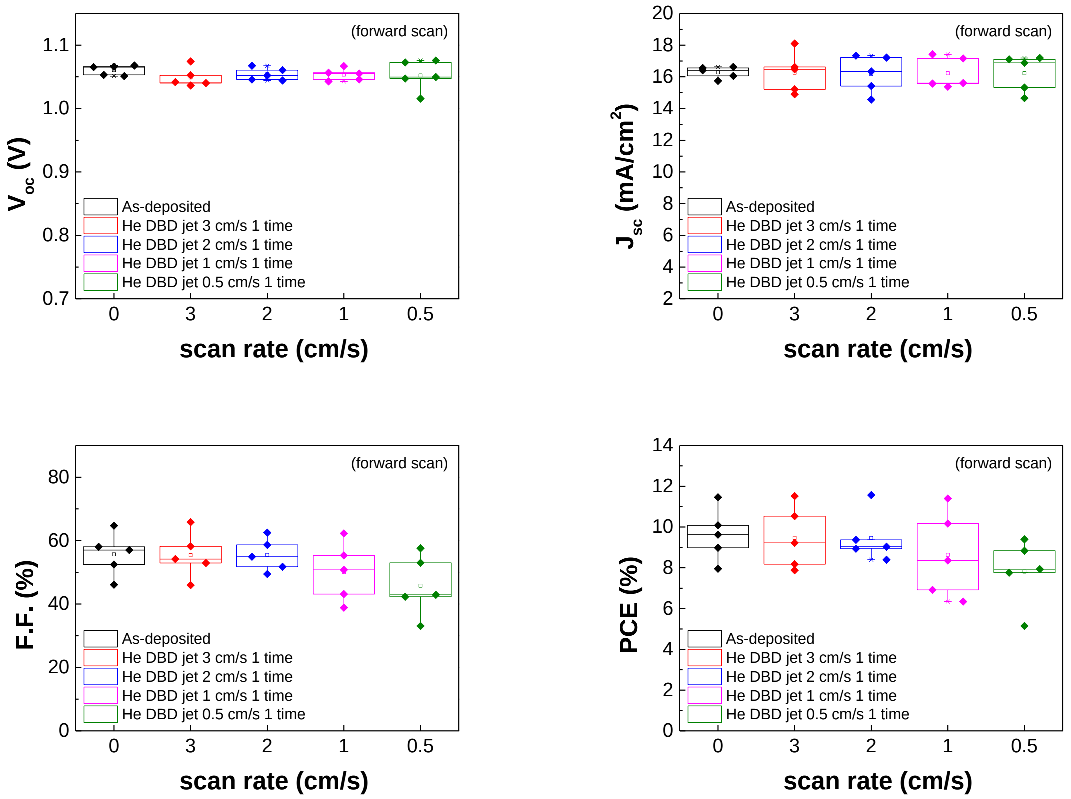

Figure S2. PV parameters of PSCs with He DBDjet treatment on BCP (forward scan)

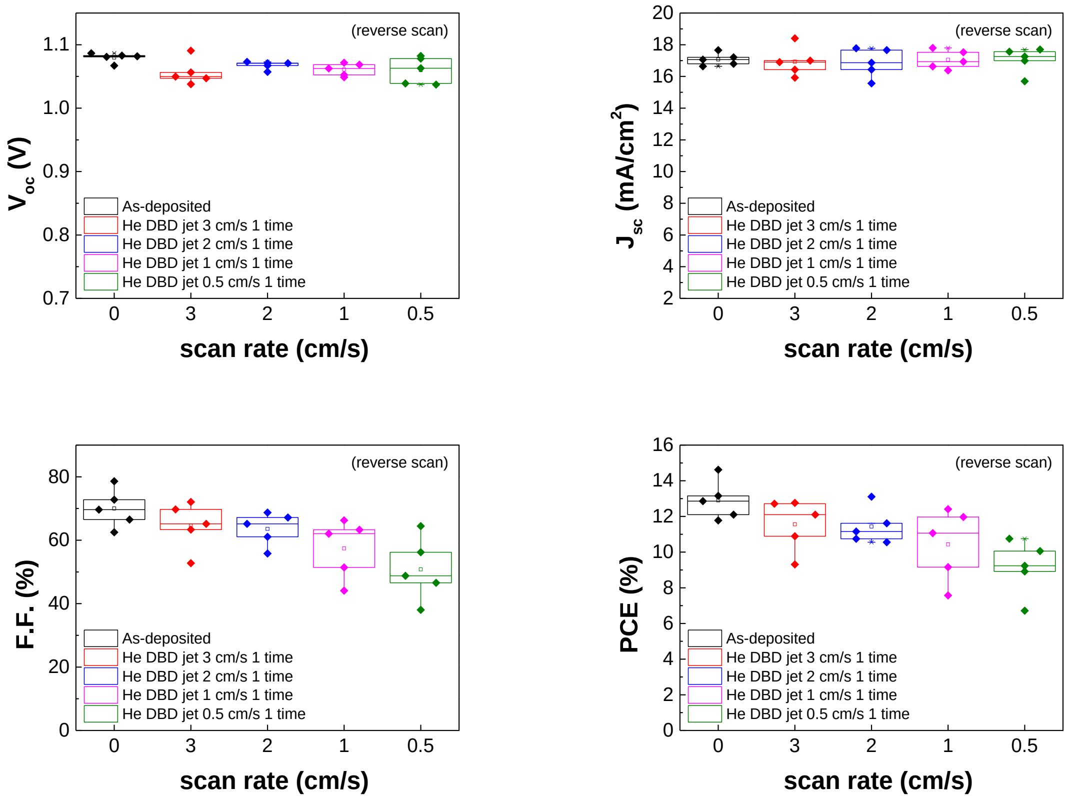

Figure S3. PV parameters of PSCs with He DBDjet treatment on BCP (reverse scan)

Table S2. PV parameters of PSCs with He DBDjet treatment on BCP

Scan rate		V_{oc} (V)	J_{sc} (mA/cm^2)	F.F. (%)	PCE (%)
No plasma treatment	Forward	1.061±0.007	16.280±0.347	55.666±9.040	9.620±1.841
	Reverse	1.080±0.007	17.073±0.584	70.007±8.593	12.901±1.719
3 cm/s	Forward	1.049±0.025	16.262±1.834	55.428±10.400	9.466±2.051
	Reverse	1.056±0.034	16.932±1.471	64.611±7.465	11.554±1.204
2 cm/s	Forward	1.054±0.013	16.175±1.160	55.463±7.038	9.460±2.105
	Reverse	1.068±0.005	16.860±0.917	63.584±5.136	11.436±1.671
1 cm/s	Forward	1.053±0.013	16.227±1.189	50.071±12.184	8.637±2.763
	Reverse	1.031±0.011	17.053±0.744	57.422±8.836	10.435±1.974
0.5 cm/s	Forward	1.052±0.024	16.230±0.955	45.772±11.801	7.814±1.579
	Reverse	1.060±0.022	17.040±0.656	50.802±13.637	9.135±1.616

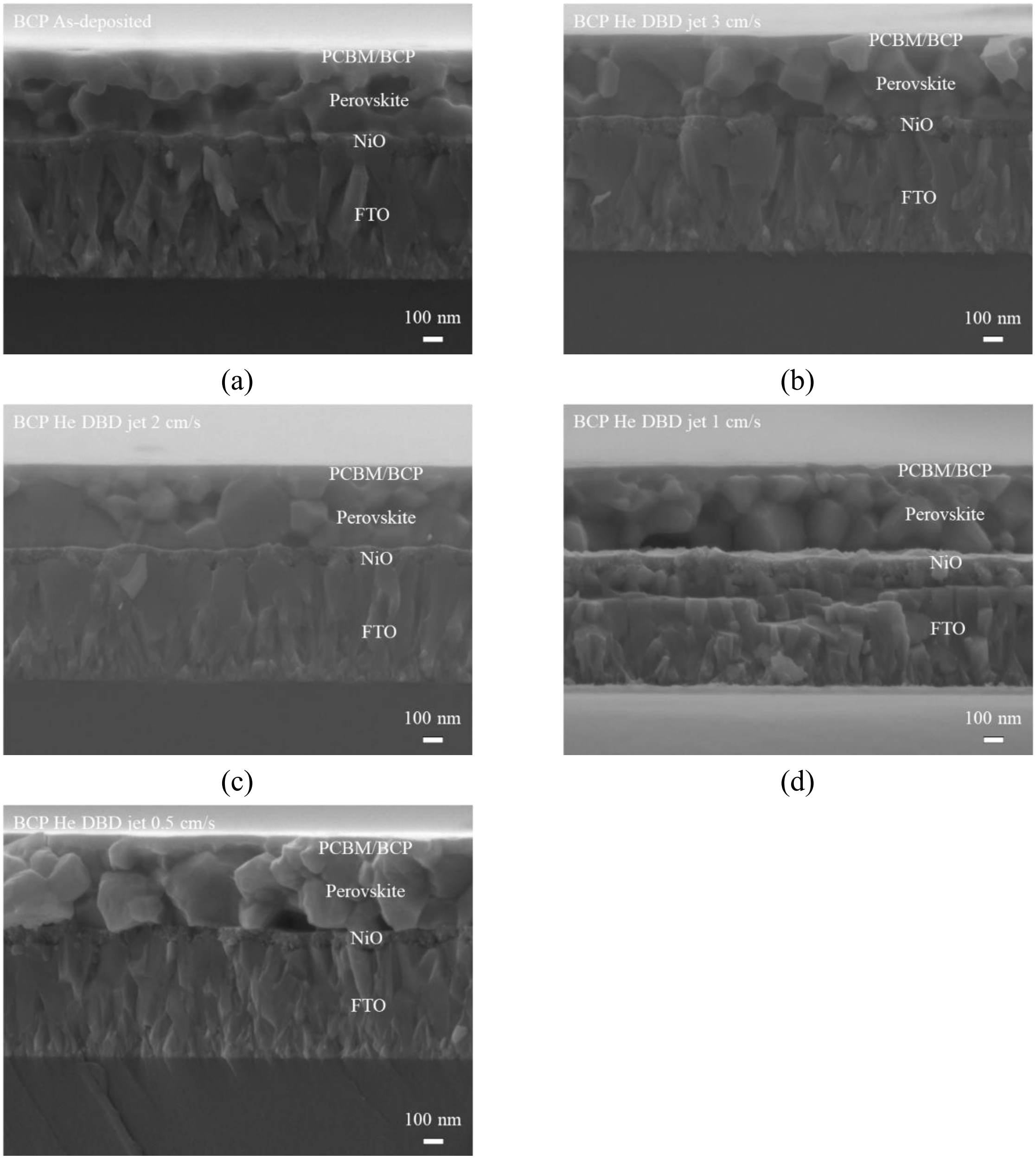

(e)

Figure S4. Cross-sectional view SEM images of the samples (a) without and (b) – (e) with DBDjet treatment under scan rates of (b) 3, (c) 2, (d) 1, and (e) 0.5 cm/s
